# Identification of a novel porcine Teschovirus 2 strain as causative agent of encephalomyelitis in suckling piglets with high mortality in China

**DOI:** 10.1186/s12917-022-03549-1

**Published:** 2023-01-04

**Authors:** Wenqi Liang, Xiangdong Wu, Zhen Ding, Shengwei Zhong, Xinjie Qian, Pei Ye, Hao Liu, Zheng Chen, Jinhua Zhang, Huabin Cao, Guoliang Hu, Junrong Luo, Zuohua Li, Nengshui Ding, Ruiming Hu

**Affiliations:** 1 Department of Veterinary Medicine, College of Animal Science and Technology, Jiangxi Agricultural University, Nanchang 330045, China; No. 1101 Zhimin Avenue, Economic and Technological Development District, Nanchang, 330045 Jiangxi China; 2 Jiangxi Provincial Key Laboratory for Animal Health, Institute of Animal Population Health, Jiangxi Agricultural University, No. 1101 Zhimin Avenue, Economic and Technological Development District, Nanchang, 330045 Jiangxi China; 3 College of Veterinary Medicine, Hunan Agricultural University, Changsha, 410128 China; 4 State Key Laboratory for Pig Genetic Improvement and Production Technology, Jiangxi Agricultural University, Nanchang, 330045 China; 5 Key Laboratory of Swine Nutrition and Feed Science of Fujian Province, Aonong Group, Zhangzhou, 363000 China

**Keywords:** Porcine teschovirus, Encephalitis, Phylogeny, Tertiary structure

## Abstract

**Background:**

Porcine Teschovirus (PTV), also named Teschovirus A, is prevalent in pig populations, mainly causing neurological symptoms, diarrhea, pneumonia, and reproductive failure, however the morbidity and mortality are usually low in pig farms.

**Case presentation:**

In this study, we reported a PTV outbreak investigation in one large-scale pig farm in China with severe symptoms including diarrhea, lethargy, locomotor ataxia, nystagmus, paralysis of the hind limbs, and coma in piglets. More importantly, the mortality reached 38% in suckling pigs, which is remarkably high in PTV history. A novel PTV strain, named HeNZ1, was isolated from cerebral samples of one suckling pig and the genome sequence was obtained by NGS sequencing. Phylogenetic and evolutionary divergence analyses revealed that HeNZ1 belongs to PTV genotype 2. Surprisingly, the VP1 coding region of HeNZ1 shares the highest sequence similarity with European PTV-2 strains, instead of China domestic PTV-2 strains, implying it may not derive from China local PTV-2 strains. Multiple sequence alignment and B cell epitope prediction of PTV VP1 and VP2 protein revealed 10 B cell epitopes, 5 mutant clusters and 36 unique mutation sites, of which 19 unique mutation sites are located in B cell epitopes and exposed on the surface of VP1 or VP2, implying significant antigenic drift potential of HeNZ1.

**Conclusion:**

These results indicate that HeNZ1 is a highly virulent PTV-2 strain, which capable of causing severe neurological symptoms and high mortality in piglets. Bioinformatic analysis suggest that HeNZ1 is genetically and antigenically different from other Chinese PTV-2 strains. Overall, current case expanded our understanding of PTV-2 clinical spectrum and revealed the emergence of a highly virulent PTV-2 strain with substantial genetic diversity and antigenic drift potential in VP1 and VP2.

**Supplementary Information:**

The online version contains supplementary material available at 10.1186/s12917-022-03549-1.

## Background

*Teschovirus A*, formerly named Porcine teschovirus (PTV), is a nonenveloped, positive-sense, single-strand, RNA virus, which belongs to the genus *Teschovirus*, and the family *Picornavirdae* [1, 2]. According to the International Committee on Taxonomy of Viruses (ICTV), PTV contains 12 validated serotypes and at least 14 genotypes, which inclines to increase with the continuous discovery of novel genotypes [1, 3–6]. The genome of PTV is approximately 7 kb with just one open reading frame (ORF), encoding the leader protein and three polyproteins (P1, P2 and P3), of which, P1 will proteolytically cleaved into VP1, VP2, VP3, VP4, and these 4 proteins form viral capsid. VP1 is the major component of viral capsid and the most diversified viral protein. Therefore, phylogenetic analysis of VP1 has been used as a reliable approach for characterizing the PTV genotypes [1]. Also, neutralizing epitopes mainly locate within VP1 and VP2 [1, 7–9].

PTV is globally endemic and circulates in most pig herds. It has been frequently detected in the feces of health pigs suggesting that these viruses could colonize in the intestinal tract without causing any disease [1, 2, 10, 11]. However, highly pathogenic PTV strains are capable of causing severe clinical signs, including severe to moderate neurological symptoms, reproductive disorders, respiratory diseases, and diarrhea [1, 2].

The first reported PTV outbreak case took place in a town named Teschen in Czech Republic 1929 with severe central nervous system disorder (CNS) in pigs [12]. Following then, PTV cases were sporadically reported in other countries, however most cases showed moderate neurological signs and low mortality [13–16]. Usually, the severe neurological symptoms caused by PTV were named Teschen disease, while the milder form of PTV associated diseases were called Talfan disease [7, 17]. In 2009, another outbreak of PTV-1 with severe CNS disorder and high mortality was recorded in the Republic of Haiti [18]. Approximately, 1500 backyard pigs were infected and the morbidity and mortality were close to 60 and 40% respectively. A case of PTV13 outbreak has reported in Spain 2017, with the morbidity of 20% and mortality of 60% in one pig farm [19].

In China, PTV associated disease in pigs was firstly reported in 2003 and a PTV-1 strain (Swine/CH/IMH/03) was isolated in this case [1]. Since then, a number of PTV strains have been sporadically discovered in multiple provinces in China, mainly associating with asymptomatic infection or milder forms of disease resulting in no more than 10% mortality rate [20]. Totally, five cases related to PTV have been reported in China since 2003 [20]. In the same year, a strain of PTV-8 (Jilin/2003) was isolated from Jilin Province in China, showing diarrhea, respiratory distress, and death in farrowing pigs [21]. In 2021, Qigai He’s group reported a PTV-1 case in the northeast China, leading to around 10% mortality in piglets [1].

In current study, we reported a severe PTV-2 outbreak in a large-scale pig farm. In this case, PTV-2 caused severe neurological symptoms and diarrhea in piglets with remarkably high morbidity and high mortality in suckling pigs. The field PTV-2 strain, named HeNZ1, in this case was isolated and the bioinformatic analysis of its genome sequences disclosed its genotype and unique mutation sites in VP1 and VP2.

### Case presentation

In June 2020, a severe disease outbreak hit a large-scale pig farm in Henan Province in China. The pig farm is a farrow-to-finish pig farms with modernized facilities and management. It contained 6000 sows and 1530 gilts at that time. In the beginning, the disease was outbreak in suckling piglets, showing severe clinical signs including diarrhea, lethargy, locomotor ataxia, nystagmus, paralysis of the hind limbs, and coma. Six days later, the disease spread to nursery piglets. The morbidity and the mortality rate in suckling piglets reached 58 and 38% respectively, while the morbidity and the mortality rate in nursery piglets were 5.4 and 0.21% respectively (Table 1). The nursery piglets being affected dominantly ranged from 21 to 60 days-old and the neurological symptoms were significantly milder. Fattening pigs, sows and gilts did not show any observable clinical signs.

**Table 1:** Morbidity and mortality rate in each subpopulation

Pig subpopulation	Total inventory	Pigs with CNS disorder or diarrhea	Morbidity	Death or succumbed pigs	Mortality
Suckling pigs	3046	1767	58%	1158	38%
Nursery pigs	19,616	1053	5.4%	42	0.21%
Fattening pigs	48,562	0	0%	0	0%
Sows	6138	0	0%	0	0%
Gilts	1530	0	0%	0	0%

Before the outbreak, the feed was not changed and no antibiotics were used, so the possibility of food poisoning and malnutrition were excluded. Given the factor that the diseases spread efficiently and the morbidity was higher than 50% in suckling pigs, we suspected that the causative pathogen might be a viral pathogen.

This study did not involve in any animal experiments. All of the tissue samples analyzed in this study were collected from piglets in the pig farm. Before their autopsy, the pigs were subjected to euthanasia by intravenous injection of sodium pentobarbital. The injection dose was 200 mg/kg. The euthanasia and autopsy were conducted by veterinarians in the pig farm.

Five suckling piglets exhibiting severe clinical signs were euthanized and the cerebral samples were subjected to PCR/RT-PCR test for diagnosis. The protocol of PCR test can be found in supplementary material and the primers used in this study were listed in supplementary material Table S1. The results showed PTV was positive in all 5 samples, while other regular viral pathogens were all negative including Porcine group A rotavirus (PARV), Transmissible gastroenteritis virus (TGEV), Porcine epidemic diarrhea virus (PEDV), Classical swine fever virus (CSFV), Pseudorabies virus (PRV), Porcine circovirus 2 (PCV2), Porcine reproductive and respiratory syndrome virus (PRRSV), African swine fever virus (ASFV), Porcine delta coronavirus (PDCoV), Porcine parvovirus 1 (PPV1), Porcine Encephalomyocarditis virus (Porcine EMCV), Porcine hemagglutinating encephalomyelitis virus (PHEV), Atypical porcine pestivirus (APPV) and Japanese encephalitis virus (JEV), Porcine sapovirus (PSaV), porcine astroviruses (PoAstV). Meanwhile, the bacteria isolation from cerebral samples and spinal cord samples were all negative.

To further confirm the PCR/RT-PCR results, cerebral samples and rectal swabs were collected from 10 suckling pigs and 5 nursery pigs showing typical clinical signs. RT-PCR test of all 30 samples were PTV positive. Also, other potential pathogens including PRRSV, PEDV, TGEV, PARV, PSaV, PRV, PHEV, APPV, JEV, PoAstV were tested and proven to be negative.

**Fig. 1:**
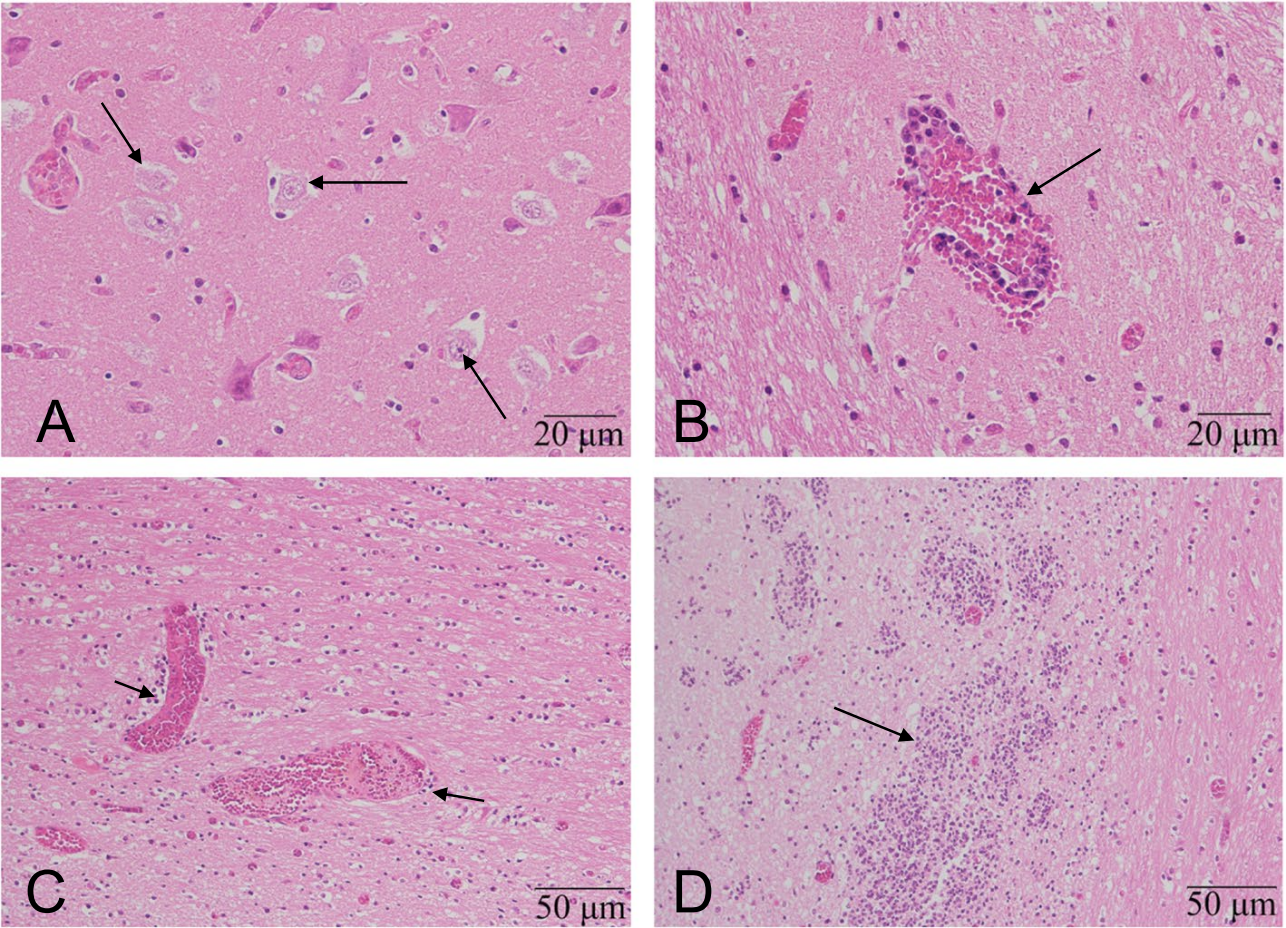
Sections of the cerebral samples from suckling pigs. Tissue sections were prepared from one piglet with severe clinical signs and stained with hematoxylin and eosin (H&E). (**A**) Neuronal degeneration and necrosis (arrow). HE. Bar = 20 μm. (**B**) The perivascular infiltrate of inflammatory cells (arrow). HE. Bar = 20 μm. (**C**) The congestion in veins. (arrow) Bar = 50 μm. (**D**) The hyperplasia of glials cells. (arrow) Bar = 50 μm.Detailed information concerning micros cope images. Microscope: Front mounted optical microscope. Objective lenses: If the bar equals 20 μm, the objective lenses is 40X, If the bar equals 50 μm, the objective lenses is 20X. Cameras: Mshot DP-MS60. Detectors: CCD. Filter model: just brightfield, no filter. Acquisition software: Image Analysis System V1.0 provided by camera supplier Mshot (https://www.m-shot.com/)

To investigate the pathological changes in CNS, cerebrum, cerebellum, and brainstem of suckling piglets were subjected for pathological evaluation. Overall, the signs of acute multifocal nonsuppurative encephalitis were found mainly in cerebellum and brainstem (Fig. 1). Neuron degeneration (neuronophagia and chromatolysis) and necrosis, along with a mild gliosis and satellitosis were observed (Fig. 1A). As shown in Fig. 1B and C, the congestion is severe and widespread, while perivascular cuffing is moderate (Fig. 1B). Pathological examination results supported the diagnosis of PTV encephalitis.

Viral isolation was applied to identify the viral pathogens in the samples by Vero cells and PK15 cells. PTV strain HeNZ1 was isolated and purified in PK15 cells and the full genome sequence of the PTV HeNZ1 was sequenced by illumina Hiseq2500 platform with 150 bp sequencing length at both terminals and the coverage >1000X. The genome of PTV HeNZ1 comprises 7209 nucleotides, which was annotated and deposited in GenBank with the accession number OL944705.

**Fig. 2:**
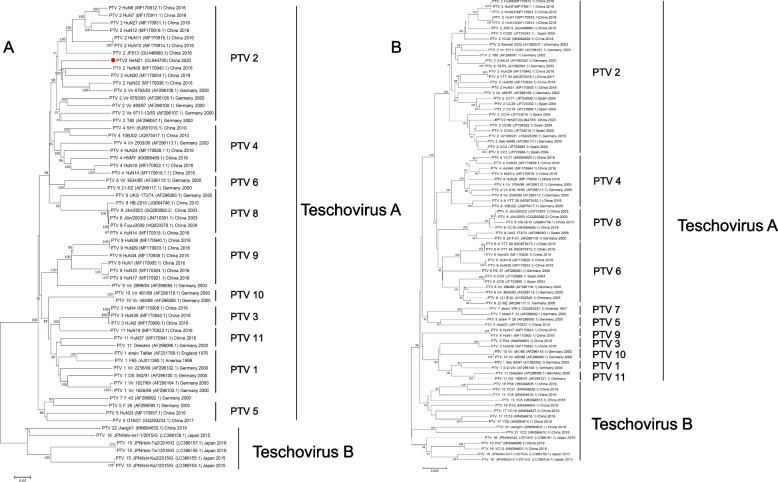
The neighbor-joining (NJ) phylogenetic tree of Porcine teschovirus. (**A**) A neighbor-joining (NJ) phylogenetic tree with 1000 bootstrap replicates was constructed in MEGA 7.0.26 based on the complete genome. (**B**) A neighbor-joining tree were constructed in MEGA 7.0.26 based on nucleotide sequences of the VP1 coding region. The reference sequences were obtained from GenBank and labeled by strain name, GenBank accession number, genotype, country origin and collection time. The scale bar represents nucleotide substitutions per site. Bootstrap values > 70% were shown. PTV strain of interest (HeNZ1) is indicated in red font

Two neighbor-joining (NJ) phylogenetic trees with 1000 bootstrap replicates were constructed based on the full genome sequences or VP1 coding regions of PTV (Fig.2A) [22]. The phylogenetic trees of both full genome and VP1 revealed that HeNZ1 clustered together with the PTV-2 strains (Fig. 2A, B). As shown in Fig. 2A, within genotype 2 clade, HeNZ1 was clustered with Chinese strains such as HuN30, HuN32, HuN39. Interestingly, although the phylogenetic analysis of VP1 coding region indicated that HeNZ1 also belongs to genotype 2, it was clustered with European strains like Sek 49/99, CC2, and CC1, while Chinese local PTV-2 strains were all clustered in other branches (Fig. 2B). Further, we performed genetic distance analysis between HeNZ1 and other strains of PTV-2 based on amino acid sequences of VP1 (Table 2). The genetic distance analyses represent the number of base substitutions per site from between sequences, which was conducted using the Poisson correction model in MEGA 7 (Table 2) [22]. HeNZ1 showed the lowest genetic distances (0.067–0.075) with a group of European PTV strains, considerably lower than the genetic distances between HeNZ1 and other Chinese PTV-2 isolates, which ranged from 0.160 to 0.173 (Table 2). Recombination analyses was performed by RDP 4.0 package with the alignment of full genome of PTV and no significant recombination event was discovered [23].

**Table 2:** Genetic distance of VP1 protein sequence of PTV-2 strains

	Strains	1	2	3	4	5	6	7	8	9	10	11	12	13	14	15	16	17	18	19	20	21	22	23	24	25	26	27	28	29
1	PTV2 HeNZ1 (OL944705)																													
2	PTV2 Sek 49/99(AF296110.1)	0.067^a^																												
3	PTV 2 CC2 (JF723985.1)	0.067	0.023																											
4	PTV 2 CC43 (JF724014.1)	0.063	0.027	0.035																										
5	PTV 2 CC44 (JF724015.1)	0.079	0.050	0.047	0.059																									
6	PTV 2 CC1 (JF723984.1)	0.071	0.027	0.004	0.039	0.050																								
7	PTV 2 CC59 (JF724022.1)	0.075	0.027	0.039	0.027	0.058	0.043																							
8	PTV 2 Vir 6793/83 (AF296108.1)	0.134	0.112	0.108	0.101	0.112	0.112	0.112																						
9	PTV 2 2-AK-III (AY392542.1)	0.138	0.087	0.100	0.101	0.100	0.104	0.104	0.055																					
10	PTV 2 12-PL (AY392541.1)	0.138	0.087	0.100	0.101	0.100	0.104	0.104	0.055	0.000																				
11	PTV 2 T80 (AF296087.1)	0.140	0.092	0.096	0.101	0.101	0.101	0.096	0.051	0.043	0.043																			
12	PTV 2 CC85 (JF724042.1)	0.142	0.134	0.125	0.135	0.138	0.129	0.138	0.116	0.100	0.100	0.101																		
13	PTV 2 Vir 480/87 (AF296109.1)	0.147	0.113	0.108	0.109	0.104	0.113	0.117	0.047	0.063	0.063	0.071	0.113																	
14	PTV 2 Stendal 2532(AY392537.1)	0.148	0.096	0.105	0.118	0.122	0.109	0.109	0.080	0.071	0.071	0.051	0.092	0.080																
15	PTV 2 Vir 6711–12/83 (AF296107.1)	0.152	0.108	0.113	0.122	0.108	0.117	0.117	0.063	0.059	0.059	0.047	0.104	0.079	0.051															
16	PTV 2 CC18 (JF723995.1)	0.153	0.135	0.135	0.140	0.131	0.140	0.135	0.076	0.105	0.105	0.105	0.140	0.084	0.131	0.101														
17	PTV 2 CC26 (JF724002.1)	0.153	0.126	0.126	0.140	0.122	0.131	0.131	0.071	0.096	0.096	0.096	0.131	0.076	0.122	0.096	0.043													
18	PTV 2 DS 183/93(AY392533.1)	0.157	0.113	0.117	0.126	0.130	0.122	0.126	0.105	0.075	0.075	0.080	0.035	0.105	0.084	0.084	0.148	0.131												
19	PTV 2 JF613 (GU446660.1)	0.160	0.121	0.121	0.131	0.129	0.125	0.134	0.125	0.096	0.096	0.101	0.063	0.117	0.084	0.104	0.166	0.144	0.043											
20	PTV 2 YC20 (MN094628.1)	0.160	0.134	0.125	0.135	0.129	0.129	0.138	0.079	0.096	0.096	0.096	0.067	0.075	0.113	0.108	0.118	0.101	0.059	0.075										
21	PTV 2 HuN10 (MF170914.1)	0.160	0.121	0.129	0.131	0.142	0.134	0.134	0.121	0.083	0.083	0.096	0.067	0.121	0.092	0.092	0.171	0.153	0.043	0.043	0.087									
22	PTV 2 HuN12 (MF170916.1)	0.160	0.121	0.129	0.131	0.142	0.134	0.134	0.121	0.083	0.083	0.096	0.071	0.121	0.084	0.092	0.166	0.148	0.047	0.039	0.091	0.019								
23	PTV 2 HuN11 (MF170915.1)	0.160	0.121	0.129	0.131	0.142	0.134	0.134	0.121	0.083	0.083	0.096	0.067	0.121	0.092	0.092	0.171	0.153	0.043	0.043	0.087	0.000	0.019							
24	PTV 2 HuN8 (MF170912.1)	0.160	0.121	0.129	0.131	0.142	0.134	0.134	0.121	0.083	0.083	0.096	0.071	0.121	0.084	0.092	0.166	0.148	0.047	0.039	0.091	0.019	0.000	0.019						
25	PTV 2 HuN27(MF170931.1)	0.160	0.121	0.129	0.131	0.142	0.134	0.134	0.121	0.083	0.083	0.096	0.071	0.121	0.084	0.092	0.166	0.148	0.047	0.039	0.091	0.019	0.000	0.019	0.000					
26	PTV 2 DS 756/93 (A63AY392534.1)	0.161	0.113	0.126	0.126	0.139	0.130	0.126	0.113	0.075	0.075	0.088	0.047	0.113	0.084	0.084	0.148	0.131	0.019	0.055	0.071	0.039	0.031	0.039	0.031	0.031				
27	PTV 2 Vir 2018/87(GQ293229.1)	0.161	0.117	0.122	0.131	0.135	0.126	0.130	0.109	0.079	0.079	0.084	0.039	0.105	0.084	0.088	0.153	0.135	0.012	0.043	0.063	0.047	0.051	0.047	0.051	0.051	0.023			
28	PTV 2 HuN7 (MF170911.1)	0.164	0.125	0.134	0.135	0.147	0.138	0.138	0.125	0.087	0.087	0.101	0.075	0.121	0.084	0.096	0.171	0.153	0.051	0.039	0.095	0.023	0.004	0.023	0.004	0.004	0.035	0.047		
29	PTV 2 HuN30 (MF170934.1)	0.173	0.138	0.138	0.144	0.129	0.142	0.142	0.083	0.117	0.117	0.101	0.108	0.096	0.122	0.117	0.113	0.101	0.105	0.129	0.083	0.134	0.134	0.134	0.134	0.134	0.113	0.109	0.138	

Note: The analysis included 29 amino acid sequences and was conducted using the Poisson correction model in MEGA 7. All positions containing gaps and missing data were eliminated. ^a^: The genetic distances displayed in this table represent the number of base substitutions per site from between sequences

**Fig. 3:**
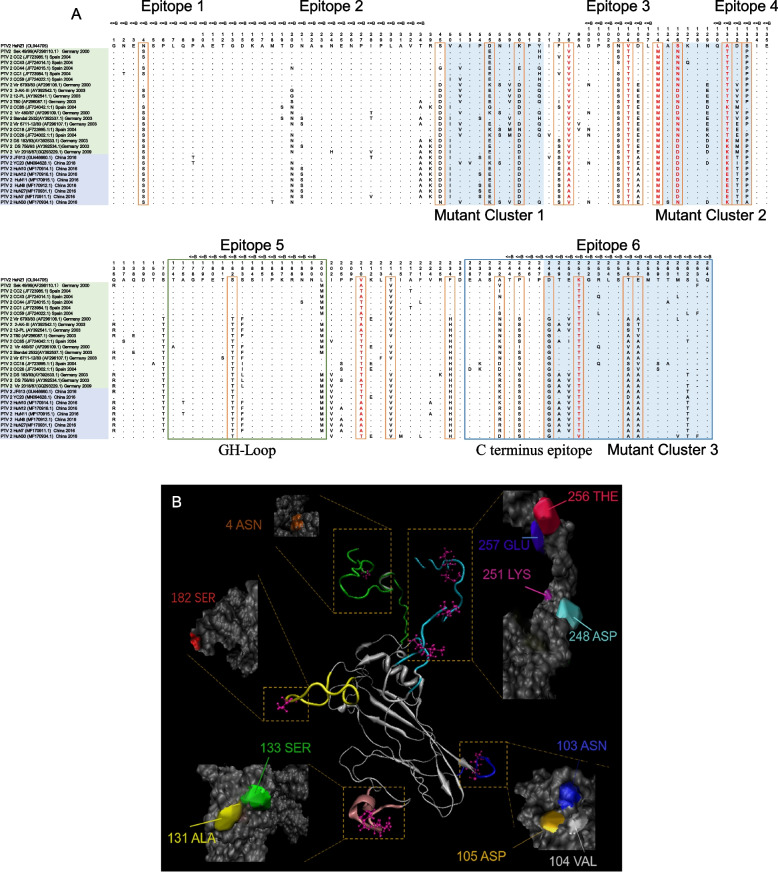
PTV-2 VP1 multiple sequence alignment and the tertiary structure prediction. (**A**) Unique mutations and mutant clusters in the VP1 coding region of HeNZ1 comparing with other genotype 2 isolates. Comparing to the other PTV-2 strains from China, 24 unique amino acid mutations in HeNZ1 VP1 were recognized and labeled with orange frame, of which 7 aa residues are unique in HeNZ1 comparing with all other PTV-2 strains and were shown in red font. Three mutant clusters are indicated by blue background. The strain name list was covered with different colors: the yellow background are the strains identified from Europe and the strains covered with purple background were identified in China. Major epitopes of PTV VP1 were identified by Disco Tope and labeled on the top of alignment. GH-loop and C terminus epitope in HeNZ1 VP1 were labeled with green frame and blue frame. (**B**) The structure of PTV VP1 were predicted using the I-TASSER online tool. Unique mutations were depicted by VMD (1.9.4a48) and the surface residues of unique mutations were highlighted

**Fig. 4:**
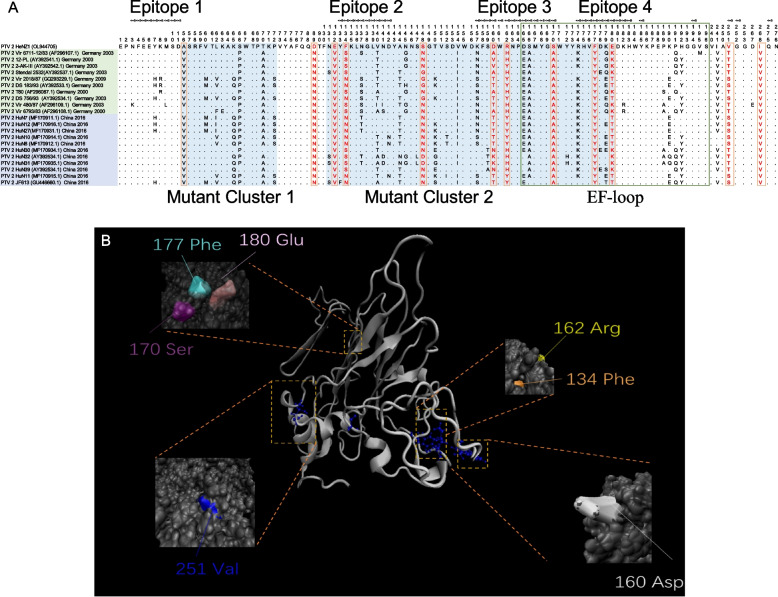
PTV-2 VP2 multiple sequence alignment and the tertiary structure prediction. (**A**) Unique mutations and mutant clusters in the VP2 coding region of HeNZ1 comparing with other genotype 2 isolates. Comparing to the other PTV-2 strains from China, 12 unique amino acid mutations in HeNZ1 VP2 were recognized and labeled with orange frame, of which 11 aa residues are unique in HeNZ1 comparing with all other PTV-2 strains and were shown in red font. Two mutant clusters were indicated by blue background. The PTV-2 strain name list were covered with different colors: the yellow background are the strains identified from Europe and the strains covered with purple background were identified in China. Major epitopes of PTV VP2 were identified by Disco Tope. EF-loop in HeNZ1 VP2 were recognized and labeled with green frame. (**B**) The structure of PTV VP2 were predicted using the I-TASSER online tool. Unique mutations were depicted by VMD (1.9.4a48) and the surface residues of unique mutations were highlighted

Previous studies showed that antigenic drift, one of the major reasons for immune evasion, were mainly recognized in VP1 and VP2 [1, 8]. Comparing to the other PTV-2 strains from China, 24 unique amino acid (aa) mutations were discovered in the VP1 region, and 12 unique aa mutations were discovered in the VP2 of isolate HeNZ1 (labeled with orange frame in Fig. 3A and Fig. 4A), of which, 7 aa residues (76I, 104 V, 114 L, 126S, 131A, 210 V, 251 K) of HeNZ1 VP1 and 11 aa residues (89D, 132E, 134F, 148S, 160D, 162R, 170S, 177F, 180E, 251 V, 268I) of HeNZ1 VP2 are unique comparing with all PTV-2 stains, which were highlighted in red font (Fig. 3A, Fig. 4A).

In this study, totally 6 B cell epitopes of VP1 predicted by using Disco Tope (http://www.cbs.dtu.dk/services/DiscoTope/) and the results were presented in Fig. 3A, along with multiple sequence alignment of VP1 [24]. As shown in Fig. 3A, multiple sequence alignment of VP1 showed 3 highly diversified regions (mutant clusters). The epitope 4 and 6 are more diversified while other 4 epitopes are relatively conservative in PTV-2, implying that epitope 4 and 6 might contribute more significantly to the antigenic drift of PTV-2 (Fig. 3A). Overall, the unique mutations in HeNZ1 VP1 located in 5 out of 6 B cell epitopes, and within the epitope 6, 5 unique aa mutations were recognized (Fig. 3A). 13 out of the 24 unique mutations sites are located within predicted B cell epitopes (Fig. 3A). In HeNZ1 VP2, 4 B cell epitopes were predicted and 12 unique mutation sites plus 2 mutation clusters were identified (Fig. 4A). According to Disco Tope results, 7 out of 12 unique mutations of HeNZ1 VP2 locate within 4 B cell epitopes (Fig. 4B). Y. Kaku et al’s work revealed several essential neutralizing B cell epitopes in VP1 and VP2 of PTV by blotting the overlap peptide library with monoclonal antibodies showing significant neutralization activity against PTV [7]. According to their results, the GH loop (aa174–201, epitope 5) and the C terminal epitope (aa236–264, epitope 6) of VP1 and the EF loop (aa165–198, epitope 3&4) of VP2 are essential neutralizing epitopes, which were labeled in Fig. 3A and Fig. 4A. As shown in Fig. 3A, one unique mutation site (T182S) is located in the GH loop of VP1 and 6 unique mutation sites (242A, 245P, 248D, 251 K, 256 T, 257E) are located within the C terminal epitope of VP1. As shown in Fig. 4A, Fig. 3 unique amino acid mutation sites (170S, 177F, 180E) are located in the EF loop of VP2. Interestingly, the C terminal epitope of VP1 and EF loop of VP2 are highly diversified while the GH loop of VP1 is relatively more conservative (Fig. 3A, Fig. 4A). All these results imply significant antigenic difference between HeNZ1 and other PTV-2 strains.

In order to better understanding the position of these unique mutation sites on the structural protein VP1 and VP2, we also predicted the tertiary structure of VP1 and VP2 by using the I-TASSER online tool [25–27] (https://zhanggroup.org/I-TASSER/). The model of VP1 was used to display the residues with a C-score of 0.31, an estimated TM-score of 0.75 ± 0.10, and an estimated RMSD of 5.3 ± 3.4 Å. The model of VP2 was used to display the residues with a C-score of 0.09, an estimated TM-score of 0.73 ± 0.11, and an estimated RMSD of 5.9 ± 3.7 Å.

Meanwhile, spDBV 4.10 (https://spdbv.unil.ch/) was used to determine the amino acid residues exposed on the protein surface (Table 3). According to spDBV results, among 13 unique mutation sites in VP1, which locate within B cell epitopes, 11 unique mutation sites are exposed to the surface of VP1 (Table 3). For VP2, among 8 unique mutation sites of VP2, which locate within B cell epitopes, 7 unique mutation sites are exposed to the surface of VP2 (Table 3). The mutation sites listed in Table 3 were depicted in the tertiary structure of VP1 and VP2 of HeNZ1 by using VMD 1.9.4a48 (Fig. 3B, Fig. 4B) [28, 29]. These results further support that PTV-2 HeNZ1 might possess significant diversity of antigen profile and antigenic drift.

**Table 3:** Antigenic epitopes and aa surface distribution of each unique mutation sites

	Amino acid residue^$^	Exposure on the surface*	Located within B cell Epitope^#^
VP1	4 N	√	√
	45S		
	55D		
	60 K	√	
	73F		
	76I		
	103 N	√	√
	104 V	√	√
	105D	√	√
	114 L		
	126S		
	131A	√	√
	132D		√
	133S	√	√
	182S	√	√
	210 V	√	
	214 T		
	234F	√	
	242A		
	245P		√
	248D		
	251 K	√	√
	256 T	√	√
	257E	√	√
VP2	134F	√	√
	160D	√	√
	162R	√	√
	170S	√	√
	177F	√	√
	180E	√	√
	251 V	√	√
	268I		√

$: The number of Amino acid residue is based on the VP1 or VP2 amino acid sequence of PTV HeNZ1. The amino acid residues listed in this table are the unique mutation sites identified in multiple sequence alignment, which were shown in Fig. 3A and Fig. 4A. a: spDBV 4.10was used to determine the amino acid residues exposed on the protein surface. #: B cell epitopes of VP1 and VP2 were predicted by using Disco Tope (http://www.cbs.dtu.dk/services/DiscoTope/) and displayed in Fig. 3A and Fig. 4A

## Discussion and Conclusion

In this current study, we reported a PTV-2 outbreak case with CNS infection and high mortality rate among suckling in a large-scale pig farm in China. The diagnosis was based on PCR results, pathological examination, and virus isolation. PCR test confirmed that all clinical samples collected from pigs with neurological symptoms were 100% PTV positive, while other pathogens were negative. In our PCR diagnostics pipeline, most viral pathogens capable of causing neurological symptoms or associating with co-infection were included.

In addition, pathologic examination showed the typical signs of nonsuppurative encephalitis indicating that the neurological symptoms were caused by a viral pathogen. Further, PTV strain HeNZ1 was isolated from clinical cerebral samples. All together, we concluded the causative pathogen of current case was PTV. Although the signs of nonsuppurative encephalitis implied a viral infection and PTV was the only viral pathogen detected by PCR test and virus isolation, it is important to recognize that not all Koch’s postulates were met in this case report. Further investigations involving animal infection experiments are necessary to characterize the virulence of PTV HeNZ1.

Viral encephalitis in pig farms can be caused by various pathogens including PRV, JEV, PHEV, PTV, APPV, PSaV, PoAstV, Porcine EMCV, of which the most prevalent pathogen in China is PRV. In current case, high fever and convulsion were not observed, which are both common symptoms in pseudorabies encephalitis [20]. Co-infection of PTV and other viral pathogens has been recorded frequently including PRRSV, CSFV, PRV, PCV-2, PSaV, PEDV and TGEV [30–32]. In current case, all common co-infection viral pathogens were excluded by PCR test.

It is worth to notice that the morbidity and mortality rates in suckling piglets were remarkably high. In the history, there were 4 recorded clinical PTV cases showing high mortality, they are 1930s in Czech with PTV-1 [7], 2009 in Republic of Haiti with PTV-1 [18], the same year in Dominica Republic with PTV-1 [18] and 2017 in Spain with PTV-13 [19]. Overall, in China, PTV associated diseases were mostly sporadic and mild and no PTV case showed mortality higher than 10% before [1, 9, 33].

Given the factor that PTV-1 was responsible for most PTV cases with severe clinical signs and high mortality, it is naturally to assume that most highly virulent PTV stains should belong to genotype 1. This is the first report that a severe PTV outbreak caused by a virulent PTV genotype 2 strain.

Phylogenetic analysis showed that HeNZ1 belongs to PTV genotype 2 (PTV-2). Interestingly, VP1 coding region of HeNZ1 is genetically closer to Spanish and German strains such as Sek 49/99, CC-1, CC-49, than China local strains, implying HeNZ1 might not derive from Chinese domestic strains. These European strains were recovered mainly from the feces samples of health pigs decades ago, indicating these strains might persistent in pig population as asymptomatic infections.

VP1 and VP2 protein both are the major components of capsid, and contain the major neutralization epitopes. The mutations in PTV VP1 may responsible for antigenic drift and immune escape. In HeNZ1 VP1, 6 B cell epitopes were predicted and 24 unique mutation sites were identified (Fig. 3A). According to Disco Tope results, 13 out of 24 unique aa mutations of HeNZ1 VP1 locate within 5 B cell epitopes, implying significant antigenic difference between HeNZ1 and other China PTV-2 viruses. Interestingly, 11 of the 13 unique mutation sites located within B cell epitopes were exposed to the surface of VP1, indicating that these mutations may responsible for the antigenic drift potential of HeNZ1 VP1 (Fig. 3B). In VP2, 4 B cell epitopes were predicted and 12 unique mutation sites were identified, of which 7 unique mutation sites were located within the predicted B cell epitopes, suggesting that VP2 also showed significant antigenic drift potential. In previous study, Y. Kaku et al. have reported 3 essential linear B cell epitopes with experimental evidence. Our results showed that unique mutations can be found in all three linear B cell epitopes, which further supported the conclusion that HeNZ1 has significant antigenic drift potential.

Overall, these results suggest substantial diversity of antigen profile and antigenic drift potential of HeNZ1. In the future, it is necessary to validate these bioinformatic prediction by evaluating the pathogenicity of HeNZ1 and the cross-neutralization capability between different PTV-2 strains in China.

In order to further understand the prevalence of PTV in China, we summarized PTV case reports and epidemiological investigation in China since 2003. Totally, 281 PTV strains were identified by epidemiological surveys or case reports [1, 9, 33–35]. Among PTV-1 to PTV-23, a total of 21 genotypes were identified in China, while PTV-4 was the most prevalent genotype in China, followed by PTV-2. Yet, only PTV-7 and PTV-23 have not been reported in China in literature [1, 9, 33–35]. The epidemiological investigation of PTV conducted by Xiuguo Hua’s group indicated that the distribution of PTV serotypes were highly diversified and uneven [35]. It also showed that PTV-4 was the most prevalent serotype found in Shanghai, followed by PTV-8 and PTV-10 [35]. Further, Taotao Yang et al. analyzed 460 samples of pig feces and 118 samples of intestinal contents from Hunan Province, all PTV genotypes were found in these samples except for PTV-7 and PTV-8. Surprisingly no dominant genotype was found in this study, indicating a high genetic diversity of PTV in Hunan pig populations [33]. It is noteworthy that current study is the first report of PTV-2 in Henan Province. Due to lack of commercial diagnosis reagents, it is reasonable to believe, PTV cases are probably more frequent than literature record, which were ignored or mistakenly recognized as other diseases in pig farms in China.

In summary, current study reported a severe PTV-2 outbreak case in pig farm in Henan Province China. A novel PTV-2 strain, named HeNZ1, was isolated from this case. The morbidity and mortality of current case were 58 and 38% suggesting that HeNZ1 is highly virulent. Bioinformatic analysis indicate that HeNZ1 may possess significant antigenic drift comparing with other Chinese PTV-2 strains. In the future, it is necessary to evaluate the virulence and antigen profile of HeNZ1 in pigs.

## Supplementary Information

**Additional file 1.** PCR diagnostic methods. The samples of Cerebral and rectal swabs were collected from the pigs showing typical clinical signs. Total RNA was extracted from each tissue sample for one-step RT-PCR analysis by using the TaKaRa Mini BEST Universal RNA Extraction Kit (TaKaRa, Dalian, China) according to the manufacturer's protocol. Total genomic DNA was extracted using TaKaRa Mini BEST Universal Genomic DNA Extraction Kit Ver.5.0. (TaKaRa, Dalian, China). The RNA viruses were detected by using the one-step RT-PCR kit Ver.2 (TaKaRa, Dalian, China). For the DNA viruses, PCR test was carried out using PrimeSTAR HS DNA Polymerase (TaKaRa, Dalian, China). The PCR products were analyzed by agarose gel electrophoresis. All the primers used were listed in Table S1.

## Data Availability

The full genome sequence of PTV HeNZ1 was deposited in GenBank with accession No.: OL944705. The NGS data of PTV HeNZ1 genome sequencing, was deposited in the GSA database of National Genomics Data Center, with the accession No. CRA009017, which can be downloaded in following link: https://bigd.big.ac.cn/gsa/browse/CRA009017. Phylogeny data, multiple sequence alignments, and other datasets used in the current study are available from the corresponding author on reasonable request.

## Abbreviation

PTV: Porcine Teschovirus
ICTV: The International Committee on Taxonomy of Viruses
ORF: Open reading frame
CNS: Central nervous system disorder
PARV: Porcine group A rotavirus
TGEV: Transmissible gastroenteritis virus
PEDV: Porcine epidemic diarrhea virus
CSFV: Classical swine fever virus
PRV: Pseudorabies virus
PCV2: Porcine circovirus 2
PRRSV: Porcine reproductive and respiratory syndrome virus
ASFV: African swine fever virus
PDCoV: Porcine delta coronavirus
PPV1: Porcine parvovirus 1;
Porcine EMCV: Porcine encephalomyocarditis virus
PHEV: Porcine hemagglutinating encephalomyelitis virus
APPV: Atypical porcine pestivirus
JEV: Japanese encephalitis virus
PSaV: Porcine sapovirus
PoAstV: porcine astrovirus
NJ: neighbor-joining
