# Supplementary material for: Identification of a novel porcine Teschovirus 2 strain as causative agent of encephalomyelitis in suckling piglets with high mortality in China
Source: BMC Vet Res. 2023 Jan 4;19:2. doi: 10.1186/s12917-022-03549-1 (PMC9810521; doi:10.1186/s12917-022-03549-1)
Supplement: Supplementary file 1 — Additional file 1. PCR diagnostic methods. The samples of Cerebral and rectal swabs were collected from the pigs showing typical clinical signs. Total RNA was extracted from each tissue sample for one-step RT-PCR analysis by using the TaKaRa Mini BEST Universal RNA Extraction Kit (TaKaRa, Dalian, China) according to the manufacturer's protocol. Total genomic DNA was extracted using TaKaRa Mini BEST Universal Genomic DNA Extraction Kit Ver.5.0. (TaKaRa, Dalian, China). The RNA viruses were detected by using the one-step RT-PCR kit Ver.2 (TaKaRa, Dalian, China). For the DNA viruses, PCR test was carried out using PrimeSTAR HS DNA Polymerase (TaKaRa, Dalian, China). The PCR products were analyzed by agarose gel electrophoresis. All the primers used were listed in Table S1. [file 12917_2022_3549_MOESM1_ESM.docx]

**Supplementary Material**

PCR diagnostic methods

The samples of Cerebral and rectal swabs were collected from the pigs showing typical clinical signs. Total RNA was extracted from each tissue sample for one-step RT-PCR analysis by using the TaKaRa Mini BEST Universal RNA Extraction Kit (TaKaRa, Dalian, China) according to the manufacturer's protocol. Total genomic DNA was extracted using TaKaRa Mini BEST Universal Genomic DNA Extraction Kit Ver.5.0. (TaKaRa, Dalian, China). The RNA viruses were detected by using the one-step RT-PCR kit Ver.2 (TaKaRa, Dalian, China). For the DNA viruses, PCR test was carried out using PrimeSTAR HS DNA Polymerase (TaKaRa, Dalian, China). The PCR products were analyzed by agarose gel electrophoresis. All the primers used were listed in Table S1.

| Table S1. Primers used in this study | | | | | |
| --- | --- | --- | --- | --- | --- |
| Pathogen | Primer Sequence (5’-3’) | | Target genes | Product size (bp) | Reference |
| PTV | F: CATTCAACTCAGCCACCAAACA  R: ATTGGAGTGCTCGAACAAGGTC | | 2c | 339 | In house build |
|  |  |  |  |  |  |
| TGEV | F: GTATAAAACCTCCTGGCTGT  R: GCCATTGATTTATGGAGACA | S | | 780 | [1] |
| PEDV | F: AGTCTTACATGCGAATTGACC  R: AGCTGACAGAAGCCATAAAGT | M | | 681 | [1] |
| CSFV | F: ATC AACCAC（A/G/T）GCATT CCTCAT（C/T）G  R: CAACC（A/G）CCATCTATCTT（T/C/A）CCACCCT | E2 | | 377 | [2] |
| PRV | F: TGCGTGTTCGTGCGCTACT  R: CCTCACGACCTGGCGTTTAT | gH | | 347 | In house build |
| PCV2 | F: CAACTGCTGTCCCAGCTGTAG  R: AGGAGGCGTTACCGCAGAAG | ORF2 | | 894 | [3] |
| PRRSV | F: GTACATTCTGGCCCCTGCCC  R: GCCCTA ATTGAATAGGTG AC | ORF6 to 3’noncoding region | | 668 | [4] |
| ASFV | F: ATG GAT ACC GAG GGA ATA GC  R: -CTT ACC GAT GAA AAT GAT AC | p72 | | 278 | [5] |
| PDCoV | F: CCCAGCTCAAGGTTTCAGAG  R: CCCAATCCTGTTTGTCTGCT | nucleocapsid protein-coding gene | | 587 | [6] |
| PPV1 | F: CACAGAAGCAACAGCAATTAGG  R: CTAGCTCTTGTGAAGATGTGG | VP2 | | 203 | [7] |
| Porcine EMCV | F: CCGTCAAGTCTTCCAACCAG  R: GCGGCTTGAACCTTCTCTATC | 3D | | 438 | [8] |
| PHEV | F: TGCACCAGGA GTCCCATCTA  R: GCCAAGTTTTGCCAGAACAA | N | | 472 | [9] |
| JEV | F: ATGACTAAAAAACCAGGAGG  R: CTTGCGAGCCACATGATTGA | E | | 350 | [10] |
| PoAstV | F: GAAKCRCTSYATGGGAARCTCCT  R: CTTTGGTCCKCCCCYCCAAA | ORF1b | | 183 | [11] |
| APPV | F: TGGGGGAAAGGGGTTAACCAG  R: ATCCGCCGGCACTCTATCAAG | E | | 275 | [12] |
| PARV | F: TTTACTCTACATAAAGCATCAAT  R: GACGGCAACTCAACCTCTCACAT | NSP4 | | 416 | [7] |
| PSaV | F: TACAGCAAGTGGGAC | Polyprotein | | 194 | [13] |
|  | R: ATGACACTGGTGAACGGCAT |  |  |  |  |

**Reference**

1. Li ZL, Zhu L, Ma JY, Zhou QF, Song YH, Sun BL, Chen RA, Xie QM, Bee YZ: Molecular characterization and phylogenetic analysis of porcine epidemic diarrhea virus (PEDV) field strains in south China. *Virus genes* 2012, 45(1):181-185.

2. Tu C, Lu Z, Li H, Yu X, Liu X, Li Y, Zhang H, Yin Z. Phylogenetic comparison of classical swine fever virus in China. Virus Res. 2001 Dec 4;81(1-2):29-37.

3. Larochelle R, Bielanski A, Müller P, Magar R. PCR detection and evidence of shedding of porcine circovirus type 2 in boar semen. J Clin Microbiol. 2000 Dec;38(12):4629-32.

4. Kim HK, Yang JS, Moon HJ, Park SJ, Luo Y, Lee CS, Song DS, Kang BK, Ann SK, Jun CH *et al*: Genetic analysis of ORF5 of recent Korean porcine reproductive and respiratory syndrome viruses (PRRSVs) in viremic sera collected from MLV-vaccinating or non-vaccinating farms. *J Vet Sci* 2009, 10(2):121-130.

5. STEAR, M. (2005). OIE Manual of Diagnostic Tests and Vaccines for Terrestrial Animals (Mammals, Birds and Bees) 5th Edn. Volumes 1 & 2. World Organization for Animal Health 2004. ISBN 92 9044 622 6. €140. Parasitology, 130(6), 727-727.

6. Lee JH, Chung HC, Nguyen VG, Moon HJ, Kim HK, Park SJ, Lee CH, Lee GE, Park BK: Detection and Phylogenetic Analysis of Porcine Deltacoronavirus in Korean Swine Farms, 2015. *Transbound Emerg Dis* 2016, 63(3):248-252.

7. Ogawa H, Taira O, Hirai T, Takeuchi H, Nagao A, Ishikawa Y, Tuchiya K, Nunoya T, Ueda S: Multiplex PCR and multiplex RT-PCR for inclusive detection of major swine DNA and RNA viruses in pigs with multiple infections. *J Virol Methods* 2009, 160(1-2):210-214.

8. Werid GM, Zhang H, Ibrahim YM, Pan Y, Zhang L, Xu Y, Zhang W, Wang W, Chen H, Fu L *et al*: Development of a Multiplex RT-PCR Assay for Simultaneous Detection of Four Potential Zoonotic Swine RNA Viruses. *Vet Sci* 2022, 9(4).

9. Rho S, Moon HJ, Park SJ, Kim HK, Keum HO, Han JY, Van Nguyen G, Park BK: Detection and genetic analysis of porcine hemagglutinating encephalomyelitis virus in South Korea. *Virus genes* 2011, 42(1):90-96.

10. Steiger Y, Ackermann M, Mettraux C, Kihm U. Rapid and biologically safe diagnosis of African swine fever virus infection by using polymerase chain reaction. J Clin Microbiol. 1992 Jan;30(1):1-8. doi: 10.1128/jcm.30.1.1-8.1992.

11. Stamelou E, Giantsis IA, Papageorgiou KV, Petridou E, Davidson I, Polizopomicronulou ZS, Papa A, Kritas SK: Epidemiology of Astrovirus, Norovirus and Sapovirus in Greek pig farms indicates high prevalence of Mamastrovirus suggesting the potential need for systematic surveillance. *Porcine Health Manag* 2022, 8(1):5.

12. Liu H, Shi K, Sun W, Zhao J, Yin Y, Si H, Qu S, Lu W: Development a multiplex RT-PCR assay for simultaneous detection of African swine fever virus, classical swine fever virus and atypical porcine pestivirus. *J Virol Methods* 2021, 287:114006.

13. Ding G, Fu Y, Li B, Chen J, Wang J, Yin B, Sha W, Liu G: Development of a multiplex RT-PCR for the detection of major diarrhoeal viruses in pig herds in China. *Transbound Emerg Dis* 2020, 67(2):678-685.
